# A Brief Review of Diagnostic Techniques and Clinical Management in Chronic Kidney Disease

**DOI:** 10.7759/cureus.49030

**Published:** 2023-11-18

**Authors:** Anant Shourya Chouhan, Meghali Kaple, Snehlata Hingway

**Affiliations:** 1 Medicine and Surgery, Jawaharlal Nehru Medical College, Datta Meghe Institute of Higher Education and Research, Wardha, IND

**Keywords:** clinical management, ckd, lifestyle modifications, pharmacological interventions, imaging modalities, biomarkers, diagnostic techniques, chronic kidney disease

## Abstract

Given its increasing incidence and detrimental effects on life expectancy and quality of life, chronic kidney disease (CKD) is a significant worldwide health concern. This review article provides a complete summary of current information on the diagnosis and management of CKD, focusing on recent advances and innovative approaches. The article discusses the most current findings on CKD risk assessment, emphasizing the need for early diagnosis utilizing better biomarkers and predictive models. A rigorous examination of diagnostic tools such as albumin-to-creatinine ratio (ACR) in urine and glomerular filtration rate (GFR) highlights their importance in determining CKD phases and etiologies. In terms of therapy, the study explores evidence-based techniques to reduce the development of CKD, such as enhanced blood pressure control, glycemic management in diabetic patients, dietary changes, and renin-angiotensin-aldosterone system (RAAS) blocking. Novel therapeutic approaches, including antifibrotic and precision medicine, are evaluated regarding their potential to revolutionize CKD treatment. The study also underlines the need for multidisciplinary therapy and patient education to achieve the best possible CKD patient outcomes. It also highlights the financial and social effects of CKD, highlighting the importance of early treatment to lower medical expenses and enhance the patient's standard of living. Finally, this review article provides a comprehensive update on CKD diagnosis and treatment, highlighting present successes alongside future potential. It is a valuable resource for healthcare professionals, academics, and policymakers who want to improve CKD treatment methods and patient outcomes.

## Introduction and background

Chronic kidney disease (CKD) has become a paramount concern for health worldwide, impacting millions of people globally, with a global prevalence estimated to be between 10.5% and 13.1% [1,2]. CKD is identified by the gradual inefficiency of kidney functioning demonstrated by an estimated glomerular filtration rate (eGFR) of 60 milliliters per minute per 1.73 m^2^, the existence of kidney damage-related symptoms for more than 90 days, or both [3]. It is characterized by the lowering of renal functioning with time, which increases the risks of dialysis, hospitalization, cardiovascular morbidity, and death [4-8]. Timely diagnosis of CKD offers a vital opportunity to avert complications and postpone the progressive loss of renal function [9-14].

The medical world has seen notable improvements in CKD diagnosis methods recently. Historically, eGFR has been used to diagnose long-term renal illness since it is regarded as a more accurate indicator of renal wellness than serum creatinine [15,16]. Today, these traditional methods have been augmented with novel biomarkers and imaging technologies that provide a deeper insight into kidney health. These innovative tools enable clinicians to identify CKD at its earliest stages, facilitating timely interventions and personalized treatment strategies. The progress of metabolomics, transcriptomics, and proteomics will lead to identifying novel biomarkers in renal illnesses, which will be made possible by the emergence of novel techniques [17]. Furthermore, practical markers must be extremely sensitive and specific for kidney disorders, correlate with the histological findings of renal biopsy and the course of the disease, and allow for the initial detection of renal impaired disorders and a favorable prognosis [18]. Once diagnosed, the clinical management of CKD requires a multifaceted and dynamic approach. Lifestyle modifications, including dietary adjustments and physical activity, are essential in disease management. Management of elevated blood pressure, renin-angiotensin-aldosterone system (RAAS) blockade, which commonly includes angiotensin-converting enzyme-1 (ACE-1) as well as angiotensin receptor blockers (ARB) for high blood pressure and albuminuria, glycemic management, and metabolic acidosis restoration are four treatments that can significantly delay the progression of CKD. Research studies have shown that treating metabolic acidosis brought on by chronic renal disease with sodium citrate or sodium bicarbonate to create regular blood pH levels delays the condition's progression [19-21]. ACE-1 or ARB medication for managing blood pressure in people with persistent renal disorders and diabetes or with no glycemia who have moderately or severely increased levels of albuminuria is backed by some evidence [22]. Furthermore, advancements in renal replacement therapies have substantially improved the standard of life and living chances for patients with end-stage renal disease (ESRD).

However, challenges persist in the optimal management of CKD. Individualized treatment plans, patient demographics, comorbidities, and genetic factors are essential for achieving the best outcomes. Additionally, integrating digital health technologies, telemedicine, and remote monitoring holds promise in enhancing care accessibility and promoting adherence to treatment regimens.

This review article comprehensively explores the contemporary landscape of diagnostic techniques and clinical management strategies for CKD. By synthesizing the latest research findings, technological innovations, and therapeutic approaches, this article aims to provide healthcare practitioners with valuable insights to effectively diagnose, manage, and ultimately mitigate the burden of CKD on a global scale.

## Review

Methodology

The methodology for the review article titled "Diagnostic Techniques and Clinical Management of CKD" involves a precise approach to gathering, analyzing, and synthesizing relevant information from existing literature through searches utilizing databases such as PubMed, MEDLINE, and Google Scholar. To find relevant papers, search terms such as "chronic kidney disease," "CKD," "diagnostic techniques," "clinical management," "biomarkers," "imaging modalities," "pharmacological interventions," and "lifestyle modifications" were employed.

To ensure that current advancements were included, the findings were selected from papers published in the last 10 years. After duplications were eliminated, abstracts and titles were evaluated for pertinent information to the review's focus on CKD diagnostic and therapy methods. Full-text publications that satisfied the inclusion criteria were carefully assessed, with significant results, methodology, and outcomes extracted. The collected literature was arranged topically, with articles classified according to diagnostic techniques, treatments, and developing trends. Throughout the study, an emphasis was placed on emerging biomarkers, sophisticated imaging methods, pharmaceutical therapies, lifestyle adjustments, and individualized care options for CKD.

**Figure 1:**
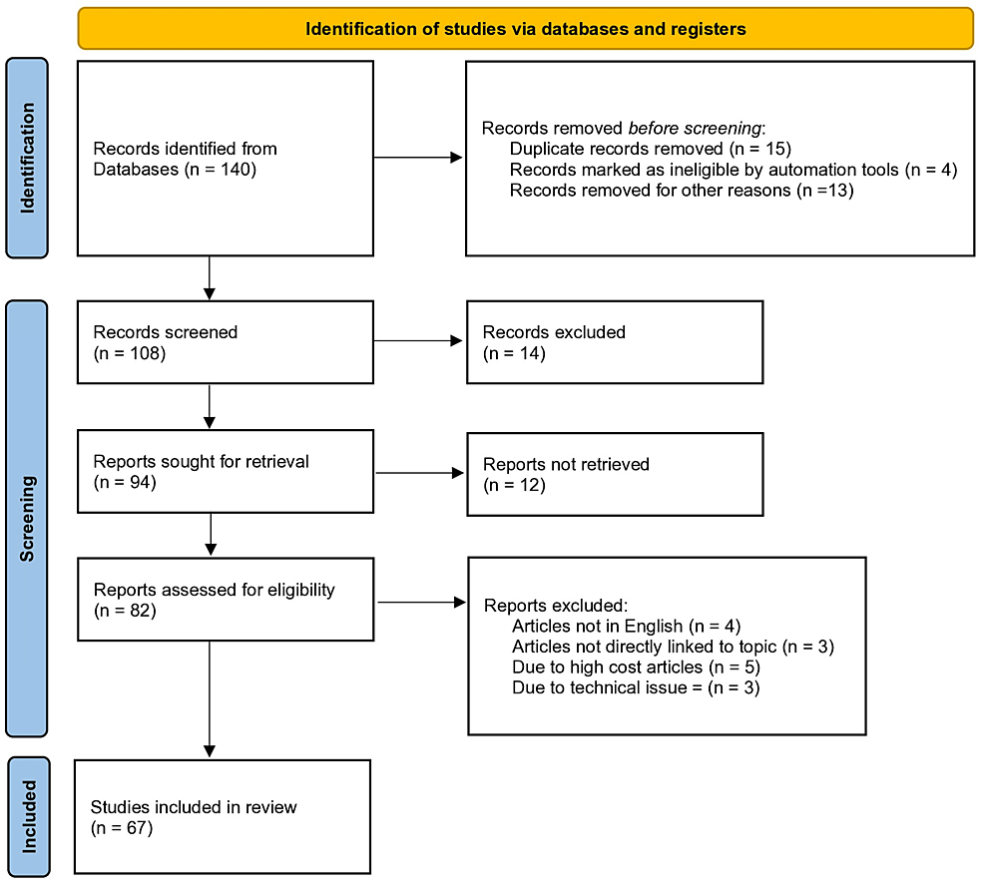
The PRISMA flowchart depicting the study selection process PRISMA: Preferred Reporting Items for Systematic Reviews and Meta-Analyses

This methodology guarantees a complete and up-to-date overview of CKD diagnostic tools and clinical care options by methodically accumulating and assessing relevant papers. The compiled data is intended to give significant insights to healthcare practitioners and academics, resulting in a better understanding of the present status of CKD care. Figure 1 depicts the Preferred Reporting Items for Systematic Reviews and Meta-Analyses (PRISMA) flow diagram detailing the study selection process.

Diagnosis of CKD

The loss of functionality of nephrons and the steady degeneration of the renal parenchyma are two characteristics of CKD [23,24]. Diagnosing CKD is a difficult task requiring a thorough grasp of many diagnostic procedures and indicators. The insidious course of CKD highlights the importance of early and precise diagnosis to reduce its impact on a patient's health and quality of life. Traditional indicators, innovative biomarkers, and sophisticated imaging modalities are used in the diagnosis procedure.

Traditional Markers

Traditional indicators are essential in detecting CKD because they provide important information about kidney function and disease progression. A severe reduction in kidney function is the initial visible indicator of the disease. Early detection (stages 1-3) can influence the development of CKD and decrease consequences [25]. Profound kidney impairment is noted in stages 4 and 5, leading to end-stage kidney dysfunction. Clinical signs of renal disease can range from minor to severe, with fast development to end-stage kidney disease. However, the intensity of clinical signs may not necessarily equate to the severity of kidney injury, as revealed by renal biopsy [26]. When compared to a biopsy, urine proteome analysis is a considerably safer choice, and it offers great promise for creating diagnostic procedures. Comparing urine analysis to blood proteome analysis, there are various benefits [27]. Because urine contains much protein, it is frequently used to search for protein indicators for various diseases [28]. Despite multiple studies, more kidney disease-specific biomarkers still need to be identified, which can be considered reliable.

The clinical evaluation of kidney function is a standard component of adult medical treatment [29]. Nowadays, most clinical labs that test blood creatinine also provide an eGFR. Serum creatinine is one of the most often used indicators to estimate GFR, representing the kidney's ability to clear waste products from the blood. It is, therefore, a crucial metric in determining the overall health of the kidneys.

Due to variance in non-GFR determinants of blood creatinine, GFR estimations remain inaccurate despite the standardization of serum creatinine tests [30]. For calculating GFR, cystatin C is considered a viable substitute for serum creatinine [31]. For people with long-term renal illness who have a poor prognosis, a test for confirmation that uses an estimated GFR based on cystatin C can be used [32].

Novel Biomarkers

Recent advancements have unveiled a range of novel biomarkers that enhance the precision and sensitivity of CKD diagnosis. These innovative markers offer insights beyond traditional parameters, enhancing diagnostic accuracy and risk stratification. Among these markers, the urinary albumin-to-creatinine ratio (UACR) has become a predictive biomarker for kidney damage and cardiovascular risk. Its sensitivity to even minor increases in albuminuria makes it an effective tool for identifying early kidney impairment and monitoring disease progression. Albuminuria is essential for determining progression despite being evaluated less commonly in clinical settings than eGFR [33]. Early kidney disease is minimally symptomatic and is typically accompanied by a regular or increased eGFR, necessitating laboratory testing. Proper diagnosis of CKD with UACR is crucial, especially in the early stages [34].

Numerous studies have been conducted on neutrophil gelatinase-associated lipocalin (NGAL) as an indicator for identifying chronic and systemic kidney disorders without bacterial infection, renal tubular damage, non-communicable systemic inflammatory response syndrome, and bacterial infections [35]. The primary sources of NGAL in the body are the loop of Henle, collecting ducts, and leucocytes [36]. Before observable changes in eGFR, urinary NGAL has been demonstrated to be a good indicator of renal injury [37].

Kidney injury molecule-1 (KIM-1) is another candidate for identifying subtle cellular damage. It is upregulated in the proximal tubules of the kidney following ischemia or any toxic damage [38,39]. Urinary KIM-1 is considered an accurate indicator of kidney damage before observable disturbances in eGFR [40]. It has been proposed as a possible biomarker of chronic renal disease caused by tubulointerstitial damage [41].

The long-lasting byproduct of proteins containing arginine, known as symmetric dimethylarginine (SDMA), is essential to basic cellular metabolic processes. SDMA is removed chiefly via the kidneys [42]. Non-renal variables such as muscle mass, nutrition, inflammation, blood sugar, and estrogen medication did not affect SDMA levels [43]. Obesity, gender, age, and polycystic ovarian syndrome all have a minor impact on SDMA [43]. Another advantage of using SDMA as a diagnostic tool is that it has less biological variability (5.8%) than its counterparts [44].

Proteomic indicators may enable more precise and early diagnosis of renal disease compared to currently used indicators like creatinine and urine albumin [45]. Since most indicator peptides in urine are produced by proteolytic activity, disease-induced alterations can be easily determined by examining proteolytic fragments [46].

Integrating these novel biomarkers complements traditional indicators, contributing to a more comprehensive assessment of CKD status. These biomarkers not only aid in diagnosis but also serve as invaluable tools for risk prediction and therapeutic monitoring. While their promise is evident, it is critical to carefully assess their sensitivity, specificity, and application to varied patient groups. Further development and confirmation of these biomarkers will be required as research continues to realize their full diagnostic potential. Overall, the introduction of novel biomarkers has heralded a new age of precision and early detection in CKD diagnosis, providing physicians with the tools they need to treat proactively and improve patient outcomes.

Imaging Modalities

Medical imaging has made an enormous contribution to clinical decision-making [47]. Imaging modalities are critical in diagnosing CKD, giving vital information on renal anatomy and function. Computed tomography (CT) scan, X-ray, magnetic resonance imaging (MRI), and ultrasonography (USG) are all methods for examining stones in patients. Compared to CT, USG has lower sensitivity for identifying renal stones [48-51]. The size, shape, and existence of structural anomalies like cysts or stones can all be learned via these imaging modalities. Its real-time imaging capabilities allow clinicians to visualize anatomical details and detect potential issues early in the disease course. MRI are often only carried out after USG or CT has identified a kidney lesion. If the enhancement during CT imaging is uncertain, an MRI may be performed after CT [52]. The prevalence of tiny, incidentally discovered kidney tumors is increasing as an outcome of the expanding utility of contemporary imaging technologies [53].

However, it is essential to balance the diagnostic benefits of imaging with potential risks, especially in scenarios where ionizing radiation is involved. The integration of imaging with other diagnostic tools, such as traditional and novel biomarkers, further enhances diagnostic accuracy and enables a holistic assessment of CKD patients. As technology advances, these imaging modalities promise continued refinement and innovation, ultimately improving our ability to diagnose and manage CKD more effectively. An illustrative table outlining the diagnosis of CKD, including standard diagnostic tests and criteria, is given below in Table 1.

**Table 1:** Diagnosis of CKD including common diagnostic tests and criteria BUN: blood urea nitrogen; eGFR: estimated glomerular filtration rate; CBC: complete blood count; ACR: albumin-to-creatinine ratio; CT: computed tomography; MRI: magnetic resonance imaging; CKD: chronic kidney disease; KDIGO: Kidney Disease Improving Global Outcomes Table Credit: Author

Diagnostic aspect	Description
History and symptoms	Inquire about symptoms (e.g., fatigue, swelling) and medical history (e.g., diabetes, hypertension).
Physical examination	Assess blood pressure, fluid status, and signs of kidney disease (e.g., edema, pallor).
Blood tests	Measure serum creatinine, BUN, eGFR, electrolytes, and CBC.
Urine tests	Perform urinalysis to assess proteinuria, hematuria, and urinary sediment. Measure ACR.
Imaging studies	Conduct renal ultrasound, CT scan, or MRI to evaluate kidney size, structure, and abnormalities.
Biopsy (if needed)	Consider kidney biopsy for the precise diagnosis and staging of CKD. Typically reserved for cases with unclear etiology or advanced disease.
Staging	Use KDIGO guidelines to categorize CKD into stages based on eGFR and albuminuria.

Challenges and Future Directions

Despite the progress in diagnostic techniques, challenges persist. In resource-limited settings, access to advanced imaging and biomarker assessments remains a barrier to early CKD detection. Moreover, incorporating novel biomarkers and imaging modalities into routine clinical practice necessitates standardized protocols and guidelines to ensure consistent and accurate interpretation. The evolving landscape of precision medicine offers promising prospects for CKD diagnosis.

In conclusion, the diagnosis of CKD has evolved significantly, encompassing a spectrum of traditional and novel biomarkers, as well as innovative imaging modalities. The integration of these techniques offers a comprehensive approach to identifying CKD at various stages and tailoring interventions to individual needs. While challenges remain, ongoing research and advancements in precision medicine hold the promise of further refining CKD diagnosis and improving patient outcomes. To deliver appropriate treatments and lessen the worldwide burden of CKD, healthcare practitioners must stay current on these discoveries.

Management of CKD

CKD is a global health challenge distinguished by its progressive nature and intricate interplay with various comorbidities. The management of CKD represents a complex and multifaceted endeavor aimed at slowing disease progression, alleviating symptoms, preventing complications, and promoting overall quality of life for individuals affected by this chronic condition. With its global prevalence steadily rising, effective CKD management strategies are paramount.

Chronic renal disease therapy aims to prevent or at least delay the disease progression and to avoid or control consequences. Management of elevated blood pressure, using RAAS blockers, diabetes management, and metabolic acidosis fixation are four therapies that have been shown to delay the progression of CKD. The following sections explain managing CKD consequences and slow progression.

Management of Hypertension

The advancement of CKD is aided by elevated blood pressure, both a cause and a consequence of the disease [54]. Afferent signals produced by functionally failing kidneys cause an increase in sympathetic tone, leading to hypertension in CKD [55]. Several factors, including an increase in oxidative metabolism and the consequent renal hypoxia, may play a role in the development of blood pressure and CKD after the initiation of hypertension [56,57]. Correct blood pressure readings are necessary for hypertension therapy to be responsive. It is advised that everyone with CKD and hypertension get treatment to achieve a target blood pressure of 130/80 mmHg or even lower, especially in diabetics [58]. Losing weight is beneficial in reducing proteinuria and blood pressure and may slow the prognosis of CKD [59]. Current recommendations advise people with chronic renal disease to consume fewer than 2000 mg of salt per day, yet there is scant data to support this advice [60].

RAAS Blockade

Pharmacological interventions constitute a cornerstone of CKD management. According to recent studies, inhibiting the RAAS can assist in managing hypertension, reduce proteinuria, and delay the progression of kidney disease [61]. The basis for dual RAAS blocking is the amalgamation of an angiotensin-converting enzyme inhibitor (ACEI) and ARB [62]. Some individuals with chronic renal illness may experience hyperkalemia or a lowered eGFR after initiating either ACEI or ARB. Only if these efforts fail should the RAAS blocker be stopped. Individuals having chronic renal illness and elevated blood pressure, regardless of diabetes status, should not get ACEI therapy and ARB therapy.

Control of Blood Glucose

Glycemic control is essential in the management of CKD, particularly in individuals with concomitant diabetes. CKD patients are more susceptible to hypoglycemia episodes and at higher risk for them [63]. It has been demonstrated that proper glycemic control can decrease the progression of CKD and reduce the mortality rate in diabetic patients. In CKD patients, non-stop blood sugar monitoring offers a choice for an accurate and thorough diabetic assessment. In addition to lowering cardiovascular risk, the benefits of diabetic control in long-term renal illness also include a decreased likelihood of albuminuria formation and a slight decrease in kidney function with time [14]. Dietary interventions, such as carbohydrate monitoring, can help stabilize blood sugar levels and prevent glycemic spikes. Medications like insulin or oral antidiabetic agents should be carefully dosed and adjusted to avoid hypoglycemia and minimize the renal burden. In conjunction with healthcare professionals, careful blood glucose monitoring enables prompt modifications to medication schedules. The progression and quality of life for people with both CKD and diabetes are eventually improved by adequate glycemic management, which is essential for slowing CKD development and lowering the risk of diabetic complications.

Correction of Metabolic Acidosis

The body's acid-base equilibrium is jeopardized by a state called metabolic acidosis, which is characterized by an acidification of the blood's pH and an excess of H^+^ ions [64]. The control and upkeep of the acid-base balance are primarily the responsibility of the kidneys, which also prevent metabolic acidosis by producing HCo_3-_ ions and flushing out extra hydrogen ions [65]. The kidney's capacity to neutralize and remove acids significantly diminishes as CKD progresses [66]. Nutritional therapy is used in conjunction with pharmaceutical therapy to address metabolic acidosis. The acid-base balance of the body is affected by the ingestion of various dietary components. Modifying food type and quality is crucial for CKD therapy. Toxin and nitrogen waste product removal is hindered in CKD patients due to renal impairment [67]. A concise table outlining the key aspects of managing CKD is given below in Table 2.

**Table 2:** Management aspects of CKD ACE: angiotensin-converting enzyme; ARB: angiotensin receptor blocker; ESA: erythropoiesis-stimulating agents; CKD: chronic kidney disease Table Credit: Author

Management aspect	Management aspect
Lifestyle changes	Encourage healthy diet, exercise, and weight management. Limit salt, potassium, and phosphorus intake. Avoid smoking and excessive alcohol.
Blood pressure control	Maintain blood pressure below 130/80 mmHg. Use ACE inhibitors or ARBs.
Blood glucose control	Optimize glycemic control in diabetic patients. Regularly monitor and adjust medications.
Medication review	Adjust drug dosages based on renal function to prevent accumulation and toxicity. Watch for adverse effects.
Anemia management	Administer ESA and iron supplements to maintain hemoglobin levels.
Fluid and electrolyte management	Monitor and manage fluid, sodium, potassium, and phosphorus levels. Adjust medications accordingly.
Regular monitoring	Conduct regular check-ups, blood tests, and imaging studies to monitor kidney function and disease progression.
Patient education	Educate patients about CKD, medications, dietary restrictions, and the importance of compliance with the treatment plan.

## Conclusions

In brief, this research delves into the intricate realm of CKD diagnosis and management. Early and precise diagnosis is pivotal in curbing CKD progression and averting complications. The diagnostic landscape is evolving, with new biomarkers like UACR, KIM-1, and NGAL supplanting traditional markers and advanced imaging techniques, providing valuable insights into kidney health. Pharmaceutical therapies, lifestyle adjustments, and the promise of precision medicine tailored to individual profiles all contribute to CKD management. Despite remarkable progress, challenges persist, including limited access to advanced diagnostics in resource-constrained settings and the need for standardized methods to incorporate novel biomarkers and precision medicine into routine clinical practice. Staying updated with the latest research is essential for navigating the complex CKD landscape. This study underscores the importance of a multidisciplinary and holistic approach to CKD management, with the overarching goal of improving outcomes and lessening the global burden of this widespread disease.
